# Arterial input function and gray matter cerebral blood volume measurements in children

**DOI:** 10.1002/jmri.25060

**Published:** 2015-10-30

**Authors:** Stephanie B. Withey, Jan Novak, Lesley MacPherson, Andrew C. Peet

**Affiliations:** ^1^ RRPPSUniversity Hospitals Birmingham NHS Foundation TrustBirminghamUK; ^2^ Birmingham Children's HospitalBirminghamUK; ^3^ Cancer SciencesUniversity of BirminghamBirminghamUK

**Keywords:** arterial input function (AIF), cerebral blood volume (CBV), dynamic susceptibility contrast (DSC) MRI, pediatric, perfusion

## Abstract

**Purpose:**

To investigate how arterial input functions (AIFs) vary with age in children and compare the use of individual and population AIFs for calculating gray matter CBV values. Quantitative measures of cerebral blood volume (CBV) using dynamic susceptibility contrast (DSC) magnetic resonance imaging (MRI) require measurement of an AIF. AIFs are affected by numerous factors including patient age. Few data presenting AIFs in the pediatric population exists.

**Materials and Methods:**

Twenty‐two previously treated pediatric brain tumor patients (mean age, 6.3 years; range, 2.0–15.3 years) underwent DSC‐MRI scans on a 3T MRI scanner over 36 visits. AIFs were measured in the middle cerebral artery. A functional form of an adult population AIF was fitted to each AIF to obtain parameters reflecting AIF shape. The relationship between parameters and age was assessed. Correlations between gray matter CBV values calculated using the resulting population and individual patient AIFs were explored.

**Results:**

There was a large variation in individual patient AIFs but correlations between AIF shape and age were observed. The center (*r* = 0.596, *P* < 0.001) and width of the first‐pass peak (*r* = 0.441, *P* = 0.007) were found to correlate significantly with age. Intrapatient coefficients of variation were significantly lower than interpatient values for all parameters (*P* < 0.001). Differences in CBV values calculated with an overall population and age‐specific population AIF compared to those calculated with individual AIFs were 31.3% and 31.0%, respectively.

**Conclusion:**

Parameters describing AIF shape correlate with patient age in line with expected changes in cardiac output. In pediatric DSC‐MRI studies individual patient AIFs are recommended. J. Magn. Reson. Imaging 2016;43:981–989

Dynamic susceptibility contrast (DSC) magnetic resonance imaging (MRI) is a method used for measuring perfusion in the brain and involves the injection of a paramagnetic contrast agent.^1^, ^2^ It provides estimates of parameters including cerebral blood flow (CBF), cerebral blood volume (CBV), and vascular mean transit time (MTT). It has been widely used in the study of stroke^3^ and brain tumors.^4^, ^5^, ^6^ In brain tumors it has been shown to be useful in tumor grading,^4^, ^7^, ^8^, ^9^ differentiating between different types of brain tumors,^10^ aiding treatment planning,^11^ assessing treatment response,^9^ differentiating between treatment effects and recurrence,^12^, ^13^, ^14^ and predicting long‐term patient outcome.^15^

The passage of injected contrast agent through the tissue results in loss of MR signal intensity, which is related to the concentration of contrast agent in the tissue. The concentration of contrast agent in the tissue is expressed as the convolution of the arterial input function (AIF) and the tissue residue function^2^—the fraction of contrast agent remaining in the tissue at time, t—modulated by CBF. Absolute calculations of CBF can be obtained by performing deconvolution of the tissue concentration–time curve and AIF and CBV can be obtained by normalizing the area under the tissue concentration–time curve by the area under the AIF.

The use of an AIF has some distinct advantages. While relative estimates of CBF and CBV can be obtained from the shape of the concentration–time curve alone, these will vary due to the shape of the AIF and the MR protocol employed. This makes comparisons between different scanners, sites, patients, and multiple visits by the same patient difficult. Pediatric studies are often, by necessity, multicenter due to small numbers of patients presenting at each center and so methods of data acquisition and analysis, including region of interest (ROI) definition, require standardization across centers. While many studies have presented brain tumor CBV values normalized to either gray^5^, ^16^ or white matter,^10^, ^12^, ^17^ this does not take into account abnormalities that may occur due to treatment. Many pediatric brain tumors occur in the mid‐line, thereby excluding the option of selecting contralateral normal brain. Normalization to gray matter is usually inappropriate in these cases. Gray matter CBVs may be of interest in their own right, for example, when assessing treatment effects on neurocognitive function. A recent study using simulated data showed that DSC parameters were highly dependent on the patient AIF and warned that parameters obtained from the shape of the concentration–time curve should be treated with caution.^18^ Other studies^19^, ^20^ have found good agreement between DSC‐MRI measures of CBF obtained using deconvolution and CBF obtained using [^15^O]H_2_O positron emission tomography (PET) and ^133^Xe SPECT, respectively, supporting the importance of using an AIF.

The AIF is a measure of the supply of contrast agent to the tissue of interest.^1^, ^2^, ^21^ It is needed in order to remove variations in the supply of contrast agent that arise due to differences in patient physiology, including cardiac output and vascular disease, as well as the injection dose and rate of administration of contrast agent during the DSC examination.^22^ Ideally, an AIF is measured close to the tissue of interest in order to minimize the effects of delay and dispersion of the contrast agent on the resulting signal–time course. An AIF is usually obtained by direct measurement of the change in contrast agent concentration during the DSC examination and requires inclusion of a suitable vessel within the MR field‐of‐view (FOV).

There are occasions when it may be difficult to measure an AIF: for example, if there is no suitable artery within the imaging FOV or where the accuracy of the AIF is in doubt. This may occur if the temporal resolution of the DSC protocol is not sufficient to satisfactorily capture the first pass peak of the AIF or if the suitable vessel within the FOV is small in comparison to the spatial resolution of the DSC time course, resulting in partial volume effects.^21^ In cases such as these, an averaged AIF obtained from a similar patient population has often been used.^23^, ^24^, ^25^ These are all based on studies of AIFs in adults and show varying differences between AIFs obtained in different patients. Due to the known variation of cardiac output with age in children,^26^ it would be expected that AIFs obtained from pediatric patients of different ages would differ significantly from each other and from those obtained in adults. Miyazaki et al^27^ presented an averaged AIF obtained from six pediatric patients scanned using dynamic contrast‐enhanced (DCE) MRI and showed differences when compared to an adult population AIF.^23^

The aims of this work were therefore: 1) To investigate the feasibility of measuring AIFs in a population of children with brain tumors undergoing surveillance DSC‐MRI scans at our hospital, and 2) To investigate how the AIFs and resulting CBV values vary with age.

## Materials and Methods

MR scans were performed on a Philips Achieva 3T TX (Philips Healthcare, Best, the Netherlands) using a 32‐channel head coil. The study was approved by the local research and ethics committee and informed parental consent was obtained.

DSC‐MRI was performed in addition to routine clinical scans that included a high‐resolution *T*_2_‐weighted TSE scan with the same coverage as the subsequent DSC scan for the purposes of defining ROIs (relaxation time / echo time [TR/TE] = 4000/100 msec). The DSC‐MRI scan was an axial FE‐EPI scan^28^, ^29^ (TR/TE = 1865/40 msec, FOV = 240 × 240 mm, matrix = 96 × 96) with a low flip angle (20°) to reduce the effect of *T*_1_ shortening in the case of blood–brain barrier breakdown^8^ while still retaining signal‐to‐noise. Thirty slices with a slice thickness of 3.5 mm each were acquired to cover the whole brain. The temporal resolution of the DSC scan was 1.86 seconds, which was repeated 60 times. Contrast agent (Dotarem, Guerbet, France) was administered via a power injector through a cannula inserted in an antecubital vein. The total dose of contrast agent given was 0.1 mmol/kg. This was given in two stages: the first half‐dose as a prebolus prior to the DSC acquisition for minimization of *T*_1_ effects^8^ and the second half‐dose at the start of timepoint 5 in the DSC data acquisition. The injection rate used was 3 mL/s, in line with recommendations from the literature.^30^ Each half‐dose of contrast was followed by a volume of up to 10 mL of saline injected at the same rate, with the volume dependent on the patient's weight.

Data were analyzed using software developed in‐house using the Python programming language (v. 2.7). The data were loaded into viewing and a suitable slice depicting the middle cerebral artery (MCA) was found. A 4 × 4 voxel square was placed on the left and right MCAs, respectively, resulting in 16 AIFs—one from each voxel in the square—for each side of the MCA. The 16 AIFs were inspected visually for shape, height, depiction of first‐ and second‐pass peaks, and suitable AIFs from each side were averaged to produce an AIF for the left and right MCAs, respectively. A gamma variate function^31^ was fit to the first pass of the left and right AIFs, respectively. The AIF used for a patient was the “best” of the left and right AIFs reflected by the lowest chi‐squared value for the respective fits.

The number of AIFs averaged in each case was noted. All AIF analyses were performed by the same researcher (S.W.). The resulting signal–time curves were converted to contrast agent concentration using:

(1) $$C\left(t\right)=-\frac{k}{\text{TE}}\text{ln}\left(\frac{S\left(t\right)}{S\left(0\right)}\right)$$

where S(t) and S(0) are the signal intensities at time, t, and baseline, respectively, TE is the time‐to‐echo of the DSC sequence, and k was assumed to be 1 for all patients. *T*_1_ effects were assumed to be negligible in analysis and the baseline signal intensity was calculated as the average signal intensity for the first six timepoints.

AIFs were interpolated to 1‐second temporal resolution and manually time‐shifted so that the last timepoint prior to the arrival of contrast agent coincided with timepoint zero removing the variation in arrival of contrast due to the time of injection and differences in bolus arrival time between patients.

Patients were separated into populations by age (2–3 years, 3–5 years, 5–10 years, and >10 years) and a population AIF for each age group was produced by averaging the AIFs of patients within each group. Patient AIFs were fitted to the Parker population AIF (Eq. (2), 23 using software written in Python, to produce estimates of parameters used to describe the shape of the AIF. The starting estimates used were those presented previously.^23^ A nonlinear least‐squares minimization routine was performed.

(2) $$C\left(t\right)=\sum_{n=1}^{2}\frac{A_{n}}{\sigma_{n}\sqrt{2\pi }}\text{exp}\left(-\frac{{\left(t-X_{n}\right)}^{2}}{{2\sigma }_{n}{}^{2}}\right)+\frac{\alpha \text{exp}\left(-\beta t\right)}{\left(1+\text{exp}\left(-s\left(t-\tau \right)\right)\right)}$$

where: A_n_ = scaling constant of nth Gaussian (mmol min); X_n_ = center of nth Gaussian (min); σ_n_ = width of nth Gaussian (min); α = amplitude of exponential (mmol); β = decay constant of exponential (min^−1^); s = width of sigmoid (min^−1^); and τ = center of sigmoid (min).

Pixel‐by‐pixel CBV values were calculated using:

(3) $$\text{CBV}=\frac{\int_{0}^{t_{\text{max}}}C_{t}\left(t\right)\text{dt}}{\int_{0}^{t_{\text{max}}}C_{a}\left(t\right)\text{dt}}$$

where C_t_(t) is the contrast agent concentration for the pixel and C_a_(t) is the AIF. CBV maps were produced for each patient using: 1) the individual patient AIF, 2) the overall population AIF and, 3) the age‐specific population AIF for that patient. ROIs were then defined in gray matter on high‐resolution *T*_2_‐weighted MR images. The ROIs were transferred to the lower‐resolution CBV maps and an average CBV for the whole ROI was computed.

### Statistical Analysis

Correlations between parameters and patient age were assessed. Each of the fitted parameters was normalized for age and intra‐ and interpatient coefficients of variability calculated using the root mean square approach. A Kruskal–Wallis test was performed to test whether the distributions of parameters were the same across each of the age groups. Statistical analyses were carried out using SPSS (Chicago, IL).

## Results

Table 1 summarizes the details of the 22 patients who underwent a total of 36 MRI scans. One patient underwent DSC scans on five visits; two patients underwent DSC scans on three visits; six patients underwent DSC scans on two visits. The mean age of the patients was 6.3 (range 2.0–15.3) years old. All except three examinations were performed under general anesthetic. Seven of the patients had undergone radiotherapy treatment prior to scanning. Four of the patients had a diagnosis of neurofibromatosis type 1 (NF1). The average total dose of contrast agent given was 4.4 (range 2.0–16.0) mL with the bolus duration of the second half‐dose given during the DSC examination ranging from 0.33–2.67 seconds. There was a significant correlation between patient age and dose of contrast agent administered (*r* = 0.803, *P* < 0.001). The average chi‐squared values of gamma variate fits to AIFs obtained from the left and right branches of the MCA were 47.7 and 35.1, respectively. The right AIF was the “better” AIF in 25 of the 36 examinations.

**Table 1:** Details of Patients Included in the Study

Tumor type	No. pts	No. scans	Mean age at date of scan (yrs)	Mean age at date of first scan (yrs)
OPG	2	3	4	4
OPG (NF1)	4	8	7	6
Pilocytic astrocytoma	3	8	8	10
Low grade glial neuronal tumors	3	4	6	7
Medulloblastoma	3	3	4	4
Other rare tumors	7	10	6	7

**Figure 1:**
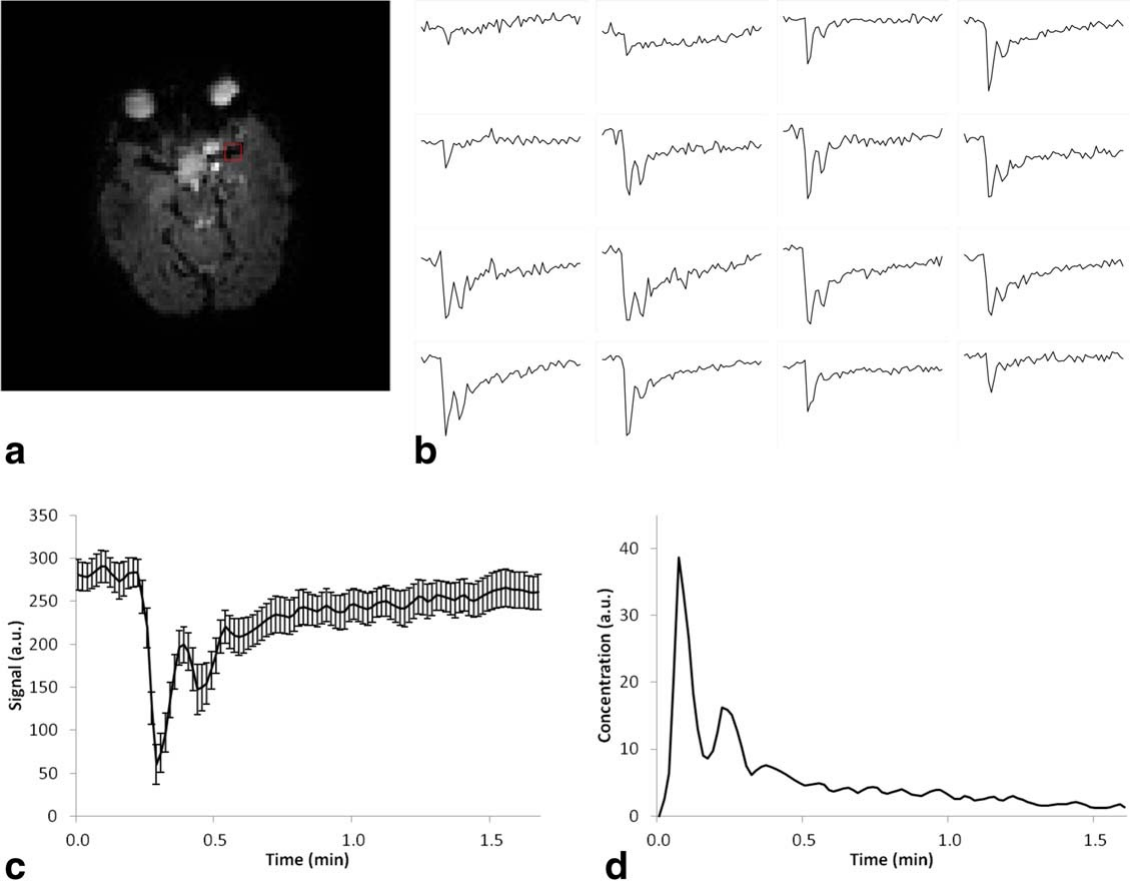
**(a)** Image from DSC‐MRI scan showing slice and location from which AIFs were obtained; **(b)** AIFs from each of the 16 voxels in the 4 × 4 voxel square selected; **(c)** Signal–time curve of AIF obtained by averaging signal–time curves from 6 voxels in the 4 × 4 voxel box. The error bars represent the standard error in the six individual pixel AIFs averaged to produce the final AIF used for this patient; **(d)** Corresponding normalized concentration–time curve interpolated to have a temporal resolution of 1 second. The patient was a 5‐year‐old girl with a pilocytic astrocytoma who received a contrast agent dose of 3 mL.

Figure 1a shows an example DSC image from a 6‐year‐old boy along with the placement of the 4 × 4 voxel box (Fig. 1a) from which AIFs from each of the 16 voxels in the box were obtained (Fig. 1b). For this patient, five of those AIFs were averaged to produce the patient AIF, which was then converted from a signal intensity–time curve (Fig. 1c) to a concentration–time curve (Fig. 1d). The AIFs for averaging were chosen manually based on shape of the signal–time curve and included only those voxels which were fully located within the MCA.

**Figure 2:**
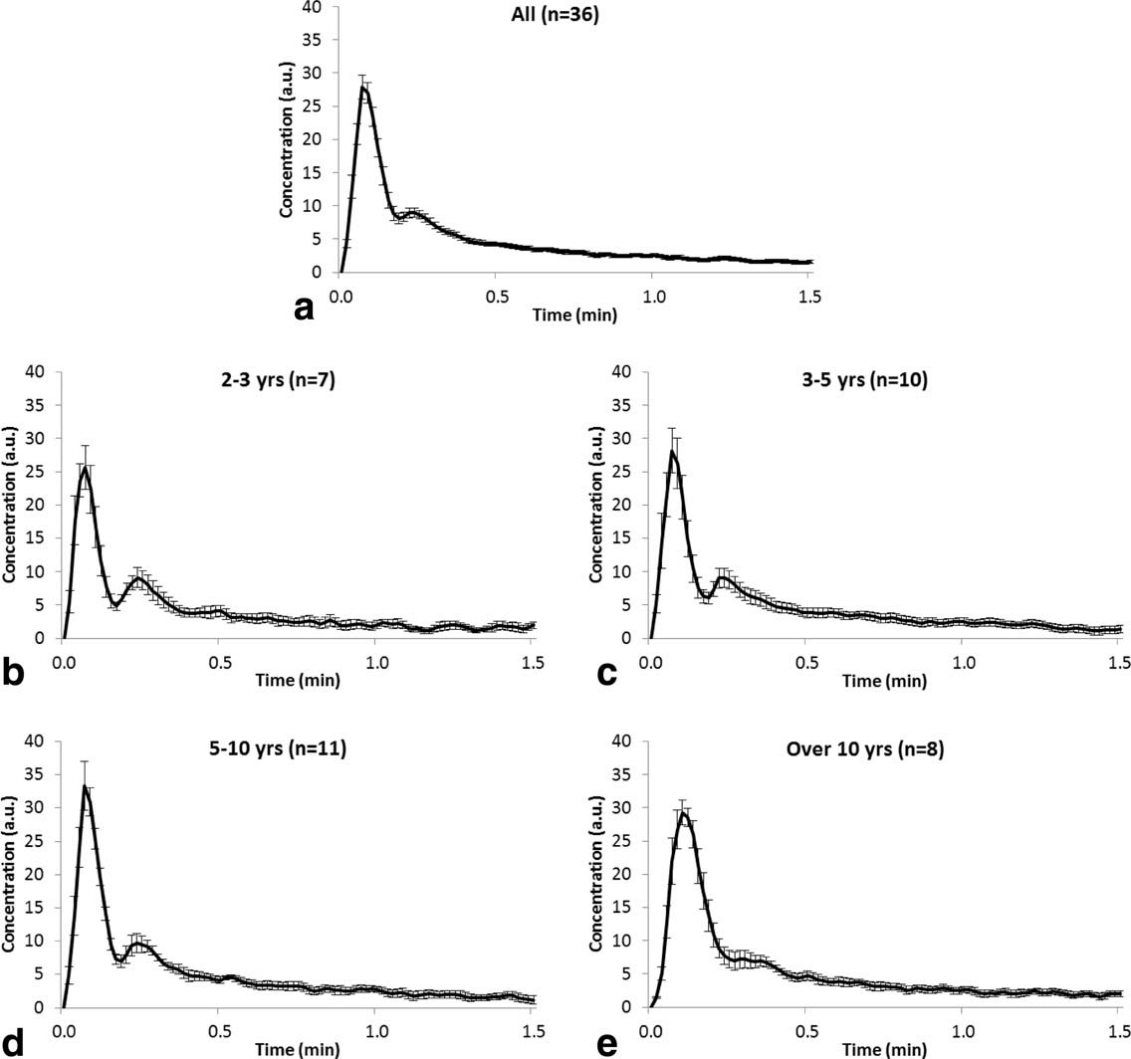
Graphs showing average normalized AIFs for: **(a)** all 22 patients scanned over 36 visits; **(b)** patients aged from 2–3 years old (*n* = 7); **(c)** patients aged from 3–5 years old (*n* = 10); **(d)** patients aged from 5–10 years old (*n* = 11); and **(e)** patients aged over 10 years old (*n* = 8). Error bars show the standard error in average AIFs for each age group. Only the first 1.5 minutes of the acquisition following the appearance of contrast in the MCA are shown to allow better visualization of the first‐ and second‐pass peaks.

**Figure 3:**
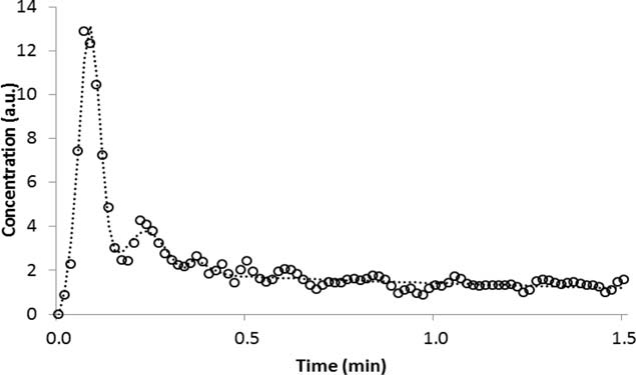
Graph showing the AIF obtained from a 6‐year‐old patient (circles) along with the fit of the Parker population AIF to the data (dotted line).

**Figure 4:**
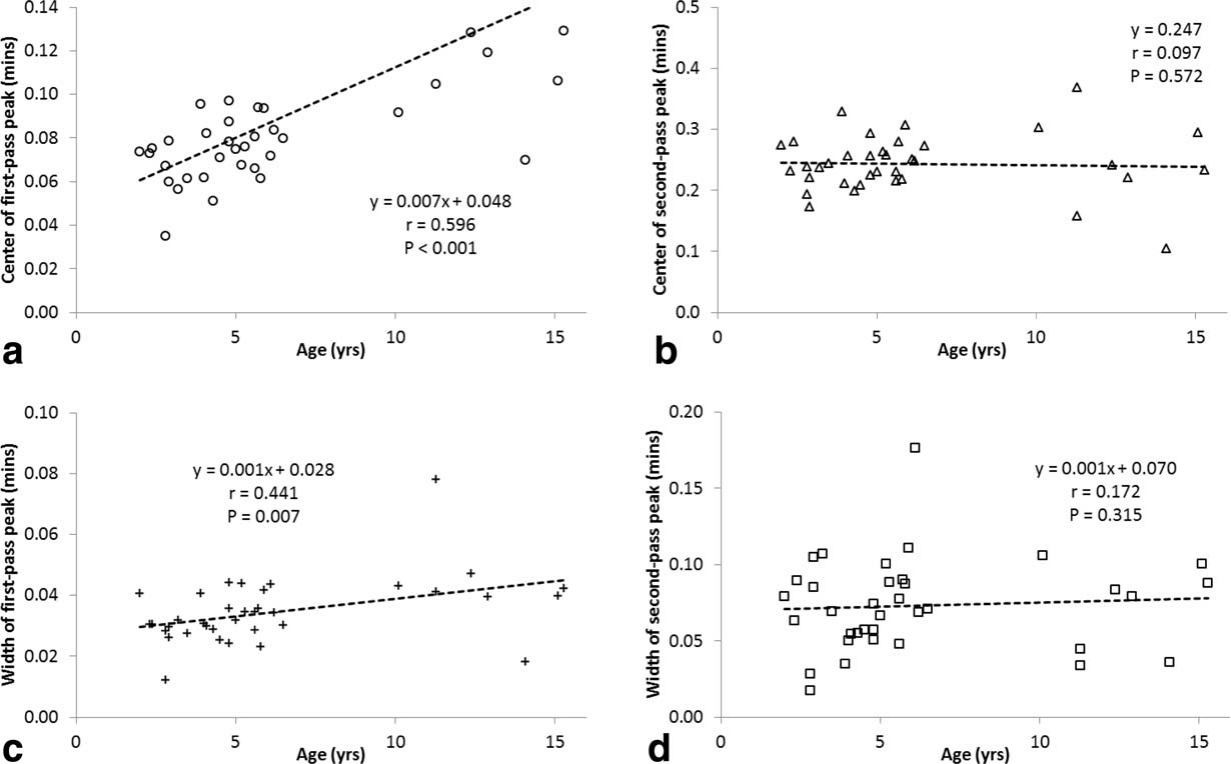
Graphs showing relationship between age and **(a)** center of first‐, X_1_; and **(b)** second‐pass, X_2_, peaks; and **(c)** widths of first‐, σ_1_; and **(d)** second‐pass, σ_2_, peaks.

Individual patient AIFs were averaged from a mean of 6 (range = 3–10) pixels. A large variation in the shape of AIFs obtained from different patients was observed. Figure 2 shows population AIFs for patients in the following age groups: 2–3 years old, 3–5 years old, 5–10 years old, and over 10 years old. A difference in the population AIFs between age groups can be observed—the population AIFs from younger patients have sharper, more pronounced first‐ and second‐pass peaks than those from the older populations. A fit of the Parker adult population AIF^23^ to a patient AIF is shown (Fig. 3). Good fits of the Parker population AIF to all patient AIFs was observed and are reflected in the chi‐squared values obtained (Table 2). The mean and standard deviation of parameters obtained by averaging values obtained from fits to all patient AIFs and over each age group are presented in Table 2. The center of the first‐pass peak was positively correlated with age (Fig. 4a; X_1_: *r* = 0.596, *P* = <0.001). The width of the first‐pass peak was significantly correlated with age (Fig. 4c; σ_1_: *r* = 0.441, *P* = 0.007). The width and center of the second‐pass peak were not significantly correlated with age (Fig. 4b; X_2_: *r* = 0.097, *P* = 0.572; Fig. 4d; σ_2_: *r* = 0.172, *P* = 0.315). The least‐squares lines of best fit for parameters with age are given in Fig. 4, allowing a population AIF to be determined at any age using Equation (2) and additional information from Table 2. A Kruskal–Wallis test showed that the distributions of the parameters X_1_ and σ_1_ were not the same across different age groups (*P* < 0.05 for both), while the distributions of other parameters with age group were not significantly different at the 0.05 significance level.

**Table 2:** Mean and Standard Deviation (SD) of Fitted Parameters and Chi‐Square Values Obtained Over All Patient Visits (*n = * 36) and by Age Group

	Mean ± SD of parameter				
Parameter	All (*n = *36)	2–3 yrs (*n = *7)	3–5 yrs (*n = *10)	5–10 yrs (*n = *11)	> 10 yrs (*n = *8)
A_1_ (mmol.min)	2.40 ± 0.92	2.01 ± 0.65	2.31 ± 0.70	2.57 ± 0.66	2.61 ± 1.53
A_2_ (mmol.min)	1.62 ± 0.62	1.55 ± 0.78	1.26 ± 0.54	1.78 ± 0.46	1.91 ± 0.62
X_1_ (min)	0.09 ± 0.05	0.09 ± 0.02	0.07 ± 0.02	0.07 ± 0.01	0.14 ± 0.10
X_2_ (min)	0.24 ± 0.05	0.23 ± 0.04	0.25 ± 0.04	0.25 ± 0.03	0.24 ± 0.08
σ_1_ (min)	0.04 ± 0.01	0.03 ± 0.01	0.03 ± 0.01	0.03 ± 0.01	0.04 ± 0.02
σ_2_ (min)	0.07 ± 0.03	0.07 ± 0.03	0.06 ± 0.02	0.09 ± 0.03	0.07 ± 0.03
α (mmol)	7.23 ± 3.80	6.78 ± 2.93	7.65 ± 5.04	7.35 ± 4.37	6.93 ± 2.12
β (min^−1^)	1.32 ± 1.39	1.40 ± 1.67	1.74 ± 1.78	1.23 ± 1.34	0.87 ± 0.38
s (min^−1^)	80.0 ± 64.7	61.6 ± 38.6	88.2 ± 81.0	75.9 ± 44.9	92.4 ± 87.5
τ (min)	0.34 ± 0.11	0.32 ± 0.11	0.31 ± 0.08	0.41 ± 0.07	0.29 ± 0.014
Chi‐sq	66.8 ± 51.4	65.7 ± 38.9	59.8 ± 40.1	90.1 ± 74.6	44.6 ± 20.4

**Figure 5:**
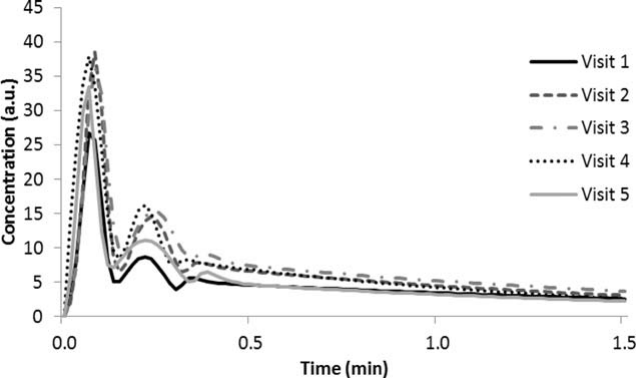
AIFs obtained from the left MCA of a patient scanned at five different timepoints following treatment for a pilocytic astrocytoma. The age of the patient at visit 1 was 5 years old. Subsequent visits were 4, 7, 14, and 16 months following the first. The patient received doses of 1.5 mL, 1.4 mL, 1.5 mL, 1.6 mL, and 1.6mL at each subsequent visit respectively.

AIFs obtained from the same patient scanned at five different timepoints are shown in Fig. 5. Intrapatient coefficients of variation were significantly lower than interpatient values for all AIF parameters (X_1_: 0.184 vs. 0.463, X_2_: 0.120 vs. 0.444, σ_1_: 0.131 vs. 0.411, σ_2_: 0.271 vs. 0.543, *P* < 0.001). AIFs obtained from the left and right MCAs showed good agreement in all patients. A paired *t*‐test performed on the left and right shape parameters obtained showed that there were no significant differences between the AIFs obtained from each side; the *P*‐values for all parameters were greater than 0.05. An unpaired *t*‐test showed no significant differences between AIF parameters or any of the CBV values measured in patients with NF1 and the rest of the population and between parameters measured in patients previously treated with radiotherapy and the rest of the population.

**Figure 6:**
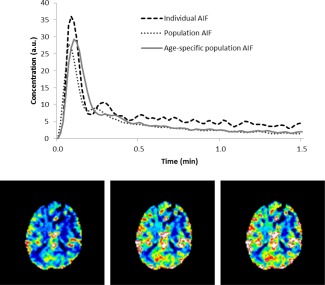
AIFs and CBV maps for a 15‐year‐old male patient with a subependymal giant cell astrocytoma. The individual patient AIF for this patient differed from the age‐specific (>10‐year group) AIF and the overall population AIF, so CBV values were very different depending on the AIF used (3.97, 5.40, 5.86 mL/100 mL for mean gray matter CBV calculated using the patient, age‐specific, and population AIFs, respectively).

Example CBV maps obtained using each of the three AIFs—patient AIF, age‐specific AIF, and overall population AIF—for a patient in the study where the individual patient AIF was quite different from the population AIFs are shown in Fig. 6. The range of gray matter CBVs observed over all patients was lower in CBVs calculated using the individual patient AIFs (mean ± SD = 5.17 ± 1.83 mL/100 mL) than using the overall population AIF (mean ± SD = 4.98 ± 2.02 mL/100 mL) and age‐specific population AIF (mean ± SD = 4.98 ± 2.01 mL/100 mL).

**Figure 7:**
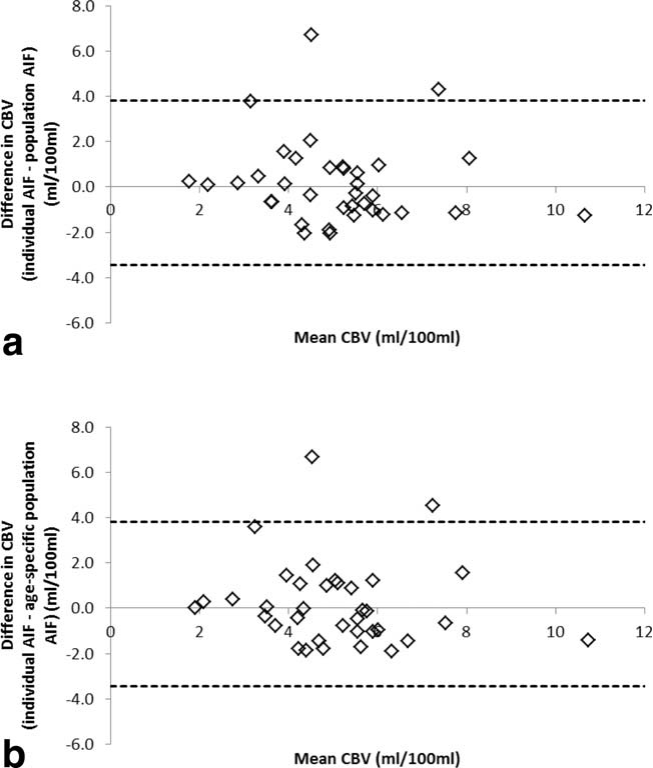
Bland–Altman plots showing the variation between CBV values calculated using the two population AIFs: **(a)** age‐specific and, **(b)** overall population, and that calculated with the individual patient AIF. 95% limits of agreement are shown.

The root mean square error in the CBV values calculated using a population and age‐specific population AIF compared to those calculated using individual patient AIFs were 31.3% and 31.0%, respectively, with the individual root mean square errors being significantly different from 0 for both the population and age‐specific population AIFs (paired *t*‐test: *P* < 0.005 in both cases). Differences in the values obtained when comparing the results obtained using an individual patient AIF and each of the population AIFs are shown in Bland‐Altman plots (Fig. 7a,b).

## Discussion

AIFs from 22 patients scanned over a total of 36 visits demonstrated a large variation in their shape, which was reflected in measures of the center and width of the first and second‐pass peaks. Fits of the AIFs to a functional form^23^ showed that the central locations and width of the first peak were significantly correlated with age. Use of these and other parameters allows an age‐specific population AIF to be determined. Intrapatient variability in AIF parameters was significantly less than interpatient variability, implying that patient characteristics play a major role in determining AIF variability.

It is well known that cardiac output—the amount of blood ejected from a ventricle in 1 minute—reduces with age.^26^, ^32^ As cardiac output lowers, less contrast will be pumped from the ventricle in each heartbeat, with the result that the contrast agent bolus becomes more spread out. As the first‐pass peak widens, the center of both peaks will shift to longer times. In addition, older, larger patients will receive higher doses of contrast agent, which along with the increased length of the cardiac system will result in longer contrast agent transit times and increased bolus delay and dispersion.

The majority of DSC studies have sought to model the first pass of contrast agent with a gamma variate function.^2^, ^18^, ^31^, ^33^ Parameters obtained when applying a gamma variate function to DSC‐MRI data obtained in 36 children aged 0.5 to 17.5 years old^33^ were shown to differ from those obtained in six adults^2^ and show that the pediatric first‐pass peak occurs almost a second before that of the adult AIF and has a width ∼4 seconds narrower than the adult AIF. The functional form of the AIF used to model the AIFs in our study was initially used for analysis of AIFs obtained in DCE‐MRI^23^ and comprises two Gaussians and an exponential modulated with a sigmoid function. The AIFs obtained in this study take the same form as those measured using DCE‐MRI; therefore, there is no reason to suggest that the model is not valid in this dataset. X_1_ and X_2_ in all age groups in our study were generally lower (X_1_: range 0.035–0.373 min; X_2_: range 0.105–0.369 min) than those presented by Parker et al in adults (X_1_: 0.17046 min; X_2_: 0.365 min).^23^ Similar patterns were observed for the peak widths (σ_1_ and σ_2_), which were lower in our study than in the adult study. A study presented by Miyazaki et al^27^ compared a population AIF obtained using DCE‐MRI in six pediatric cancer patients with those presented in the adult study.^23^ Their results agree well with ours: the pediatric population AIF was found to have a more rapid, narrower first‐pass peak and more clearly defined recirculation peak than the adult AIF.

Quantitative estimates of CBF, CBV, and MTT in DSC‐MRI require measurement of an AIF and have been shown to be more accurate than the same parameters calculated from the shape of the concentration–time curve alone.^18^ While relative estimates of these parameters are of use in brain tumor studies,^5^, ^10^, ^12^, ^16^, ^17^ quantitative estimates allow comparison between patients in multicenter studies, between scans obtained on different visits and different scanners,^18^ and can be used in assessing gray matter CBV. The large variation in AIFs observed between patients suggests that patient AIFs should be measured on an individual basis wherever possible, in agreement with other studies.^22^, ^25^

Mean CBV values in adult gray matter have been shown to range by between 3 and 7 mL/100 mL,^34^, ^35^, ^36^, ^37^ although data analysis methods vary across these references. No systematic errors in CBVs calculated with the respective population AIFs were observed when compared to those calculated with individual AIFs. Average differences of ∼30% were observed, however, with the largest error reaching 70%. An error in CBV of this magnitude is unacceptable when looking at changes in CBV values with treatment which may be significantly smaller than the error due to the AIF used. Gray matter CBVs in our study were also found to have a smaller range when calculated using individual AIFs. Taken together, these results suggest that variations in patient AIF should be taken into account when calculating CBV.

There are a number of potential limitations to this study. The majority of our patients were scanned under general anesthetic (GA). While studies suggest that the drugs given may affect cardiac output,^38^ no significant difference between AIF parameters in the GA and non‐GA groups was observed. The children included in the study have a variety of brain tumors and have undergone various treatments for their condition, both of which may affect the vasculature, for example, radiotherapy and NF1.^39^ We observed no significant differences between parameters in the small number of patients with either NF1 or those treated with radiotherapy.

Ideally, an AIF would be obtained from the artery that directly supplies the tissue of interest, known as a local AIF; however, supplying arteries tend to be small, resulting in partial volume effects. In this study, a global AIF was obtained from the left and right MCAs, and the most appropriate AIF chosen. While the shape of the AIF may be the closest to that expected, there is no guarantee that this AIF is the most suitable AIF for subsequent analysis of the DSC data, or that the AIF is not affected by the abnormality in some way. The placing of the box over the MCA and subsequent selection of suitable AIFs for averaging were performed manually by the same user for consistency. Studies have presented a variety of automated methods^40^, ^41^ for obtaining AIFs, which use software to include or exclude voxels based on their shape. While these remove the user‐variability of the method, errors can still arise if individual AIFs are not checked for shape and location.

The number of patients included in the study was small, resulting in large errors in the population AIFs and resulting parameters. However, sufficient data exists to ascertain significant differences in the parameters with age and between some of the parameters across the different age groups, as reflected in the results of the Kruskal–Wallis test. Variations in age‐normalized parameters across visits by the same patient were lower than the variation seen between patients, suggesting good reproducibility of AIFs. This suggests that, once available, a patient's AIF could potentially be used in future studies on that patient.

In conclusion, an AIF is necessary to obtain absolute estimates of CBF and CBV in DSC‐MRI studies. We showed that AIFs obtained in pediatric brain tumor patients vary in shape and that parameters describing the shape of the AIF show a correlation with age. The large variations seen, good reproducibility of individual patient AIFs, and resulting CBV values obtained suggest that measurement of individual patient AIFs is preferable to using a population AIF. Where individual patient AIF measurement is not possible, a pediatric population AIF could be used.
